# Duodenal Carcinosarcoma: An Apple-Core Lesion Causing Gastric Outlet Obstruction

**DOI:** 10.7759/cureus.58725

**Published:** 2024-04-22

**Authors:** Eli A Zaher, Mohamed A Ebrahim, Yasmin Gerais, Parth Patel, Omar Al Salman

**Affiliations:** 1 Internal Medicine, Ascension Saint Joseph - Chicago, Chicago, USA; 2 Gastroenterology, Ascension Saint Joseph - Joliet, Joliet, USA

**Keywords:** sarcoma, carcinoma, biopsy, computed tomography, carcinosarcoma

## Abstract

We present a case of a 58-year-old male with a rare duodenal carcinosarcoma causing gastric outlet obstruction. Despite its aggressive nature and poor prognosis, with only 12 documented cases in the literature, this report sheds light on the clinical presentation and challenges in diagnosis and treatment. Carcinosarcoma, characterized by both carcinomatous and sarcomatous elements, poses difficulties in management due to its diverse tissue characteristics. Surgical resection remains the primary treatment, although the prognosis remains grim, emphasizing the need for further research into advanced therapeutic strategies to improve patient outcomes. This case underscores the rarity and clinical complexities associated with duodenal carcinosarcomas.

## Introduction

Carcinosarcomas, a form of malignant tumor, are characterized by the presence of both carcinomatous and sarcomatous elements with intertwined growth patterns. The term "carcinosarcoma" was coined by Meyer, following Virchow's initial report of sarcoma carcinomatoides in 1864. While this tumor can manifest in multiple organs like the uterus, lung, and hepatobiliary tract, occurrences in the duodenum are exceptionally rare, with only 12 cases documented in the literature [1,2].

## Case presentation

A 58-year-old male presented to the emergency department with complaints of epigastric pain and non-bloody vomiting for two weeks. The pain was postprandial with radiation to the back. His history was significant for compensated cirrhosis, chronic hepatitis C, and alcoholic pancreatitis. He was noncompliant with medications.

Upon admission, vital signs were consistent with tachycardia to 111 beats per minute but were otherwise normal. The physical exam was unremarkable. Blood work-up, including liver function panel and lipase, was negative. Urine toxicology was positive for cannabis and opiates.

**Figure 1:**
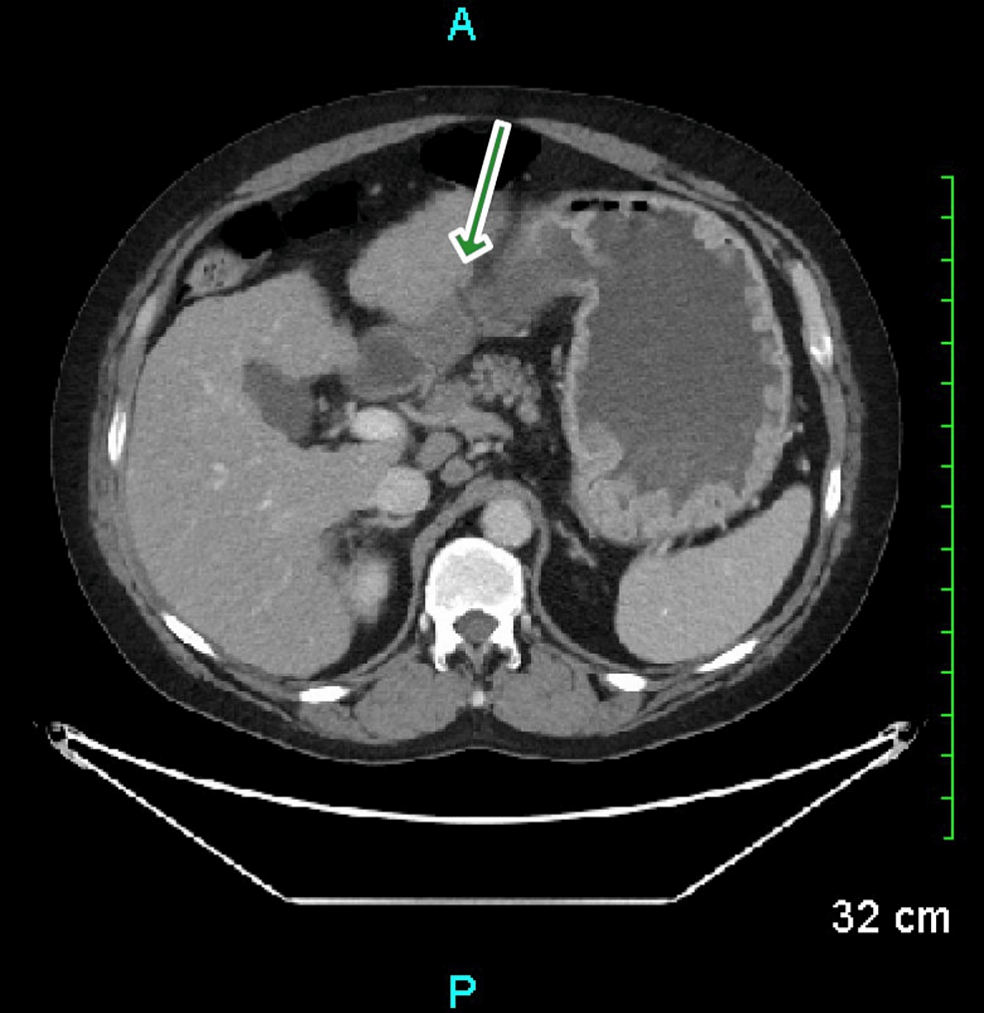
CT Abdomen The green arrow pointing towards an apple-core lesion in the duodenum.

Computed tomography (CT) of the abdomen showed an abnormal apple core-type wall thickening and associated luminal narrowing in the second portion of the duodenum (Figure 1).

**Figure 2:**
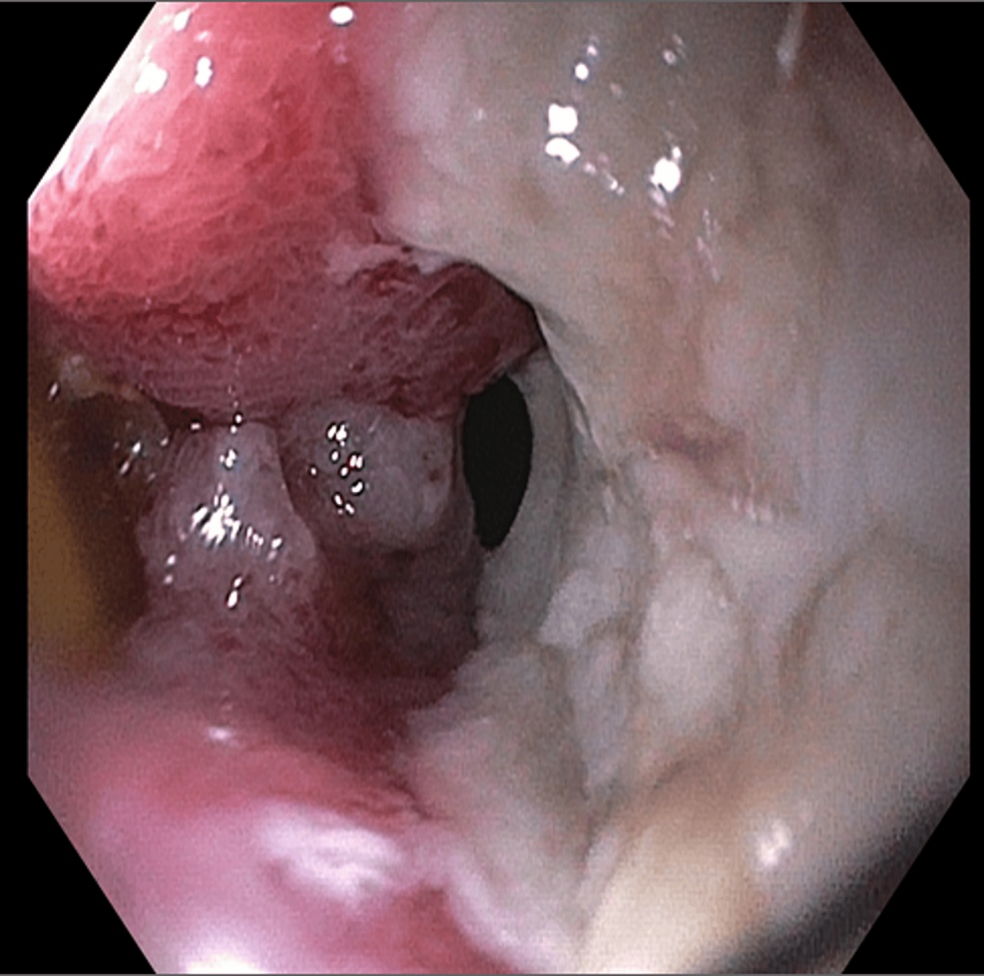
The Duodenal Bulb As Seen Through Upper Endoscopy

Gastroenterology performed an upper endoscopy which showed a large ulcerated mass in the duodenal bulb, which was biopsied with cold forceps (Figure 2).

**Figure 3:**
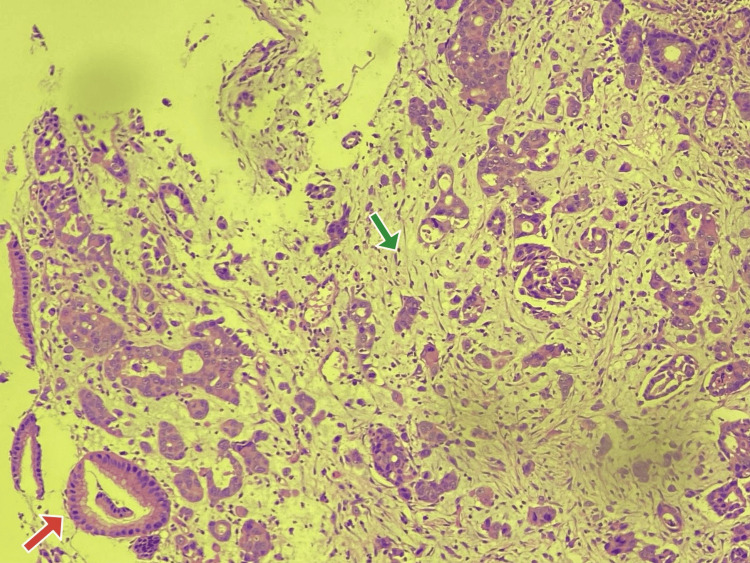
Histology of the Duodenal Mass The red arrow pointing towards the carcinoma component. The green arrow pointing towards the sarcoma component.

Given the large mass, the adult esophagoduodenoscopy (EGD) scope could not enter the second portion of the duodenum. Histology was positive for carcinosarcoma (Figure 3).

The patient was planned to be transferred to a tertiary care center for a multidisciplinary approach. He, however, refused and was lost to follow-up.

## Discussion

Carcinosarcoma is a biphasic tumor characterized by a mixture of epithelial (cancerous) and non-epithelial (sarcoma) components within a single lesion. The sarcomatous parts of carcinosarcoma often exhibit various tissue characteristics such as bone, cartilage, muscle, or fat differentiation. Those lacking clear differentiation are termed "true carcinosarcomas," showing varied interstitial tissue differentiations like rhabdomyosarcoma, leiomyosarcoma, chondrosarcoma, and osteosarcoma. In these cases, the sarcoma-like tissue primarily consists of spindle-shaped cells without distinct interstitial tissue formation. True carcinosarcomas exhibit three main features: a genuine sarcomatous component, absence of a transitional zone between carcinoma and sarcoma, and positivity for mesenchymal markers while being negative for epithelial markers. However, there's debate regarding whether all these histological criteria must be met for a diagnosis of true carcinosarcoma. In a particular case, the tumor predominantly comprised sarcomatous components with spindle-shaped cell proliferation and cytokeratin, leading to a diagnosis of a so-called carcinoma [3]. This aggressive tumor arising from the duodenum, as evidenced in our case, manifested with gastric outlet obstruction.

Carcinosarcoma has been documented in various organs including the uterus, ovary, gastrointestinal tract, pancreas, bile duct, liver, lung, and breast [4]. Duodenal carcinosarcoma, specifically, is exceptionally rare, with only 12 reported cases to date [2]. The precise mechanism underlying its development remains unclear, though several theories have been proposed, including the collision theory where mesenchymal and epithelial stem cells give rise to separate tumors, the composition tumor theory suggesting the sarcomatoid component reacts to carcinoma invasion, the metaplastic tumor theory proposing sarcomatoid changes in carcinoma lead to its development, and the combination tumor theory suggesting each component originates from a single common stem cell. Recent studies investigating p53 mutation and loss of heterozygosity have supported the monoclonal hypothesis [5].

Achieving a complete cure for carcinosarcoma of the ampulla of Vater necessitates en-bloc resection along with lymph node dissection. This type of tumor often involves lymph node metastasis, highlighting the importance of lymph node dissection during resection [2]. Similarly, carcinosarcoma in other organs is associated with poor prognosis, with many patients succumbing to liver metastases within a year post-surgery. According to Okabayashi et al., the overall survival rates at one-, two-, and three-years post-surgery for carcinosarcoma of the gallbladder, the most common site within the biliary tract, were 37.2%, 31.0%, and 31.0% respectively [6]. However, some cases may achieve long-term survival following resection, emphasizing the importance of aggressive resection with curative intent when feasible [7]. Surgery remains the primary treatment modality for carcinosarcomas, as a trial of radiotherapy and chemotherapy offered no survival benefit for carcinosarcoma of the biliary tract [8]. Due to the mingling of epithelial and non-epithelial components, traditional chemotherapy has shown limited success in treating metastasis and relapse [9].

## Conclusions

The rarity of carcinosarcoma in the duodenum highlights the significance of our case, providing valuable insights into its clinical presentation. This case report highlights an exceedingly rare occurrence of carcinosarcoma in the duodenum, presenting with gastric outlet obstruction, emphasizing its aggressive nature and poor prognosis akin to other sarcomas. Mainstay treatment necessitates en-bloc resection along with lymph node dissection for hopeful curative outcomes. However, the daunting prognosis linked with this malignancy underscores the urgent requirement for ongoing research aimed at developing advanced therapeutic approaches, promising enhanced patient outcomes in the coming years.
